# Supplementary material for: Dynamic transcriptomic profiles of zebrafish gills in response to zinc supplementation
Source: BMC Genomics. 2010 Oct 11;11:553. doi: 10.1186/1471-2164-11-553 (PMC3091702; doi:10.1186/1471-2164-11-553)
Supplement: Additional file 2 — Interactive Direct Interaction Network representing the molecular interactions between zinc, copper, iron, calcium and proteins encoded by transcripts changed by zinc supplementation. Mini web-site containing index.html and hyperlinked pages in subdirectory describing a Direct Interaction Network automatically generated based on curated interactions contained within the proprietary PathwayArchitect database. Ovals represent proteins and the circles symbolize metal ions. Objects are coloured by their abundance in zebrafish at the time-point they were significantly different from the control is a scale from -4 fold (dark green) to +4 fold (dark red). Where significant differences were found at more than one time-point, the colour overlay shows expression at the first instance. Dark blue squares denote 'binding', and light blue squares 'expression'; green squares stand for 'regulation', green diamonds for 'metabolism', and green circles for 'promoter binding'. Arrow heads indicate directionality of the interaction where annotated. All nodes and edges can be further interrogated by selecting the relative area of the image. [file 1471-2164-11-553-S2.zip › PathwayArchitect Zn xs DIN/602808.html]

# REGULATION:

|  |  |
| --- | --- |
| Type | REGULATION |
| Effect | Positive |


---

|  |  |
| --- | --- |
| Score | 0 |


---

|  |  |
| --- | --- |
| Reference Count | 5 |


---

|  |  |
| --- | --- |
| Mechanism | Unknown |


---

|  |  |
| --- | --- |
| Reference:0 || Sentence | "Using promyelocytic HL-60 cells, we have observed an exquisite sensitivity to Ca2+ of c-fos, c-jun, and zif268 mRNA accumulation, since early and maximal inductions were observed at 200-300 nM [Ca2+]i." |
| PMID | 8344941 |
| Year | 1993 |
| Species | Human |
| Journal | J Biol Chem |
| RefScore | 1 |
| Source | PArchNLP |
  |
|  |  |


---

|  |  |
| --- | --- |
 Reference:1 || Sentence | "The effect of L-glutamate (L-Glu) and its structural analogs N-methyl-D-aspartate (NMDA), quisqualate (QA), and kainate (KA) on the DNA binding activity of the Activator Protein 1 (AP-1) and the Ca2+/cAMP Responsive Element Binding Protein (CREB) families of transcription factors was examined in cultured chick retinal M�ller glia cells." |
| PMID | 7650753 |
| Year | 1995 |
| Journal | J Neurosci Res |
| RefScore | 3 |
| Source | PArchNLP |
  ||  |  |


---

|  |  |
| --- | --- |
 Reference:2 || Sentence | "In contrast, only AP-1 sites contributed to Ca(2+)-mediated VEGF-Luc induction." |
| PMID | 11865053 |
| Year | 2002 |
| Species | Human |
|  | Mouse |
| Journal | Mol Cell Biol |
| RefScore | 0 |
| Source | PArchNLP |
  ||  |  |


---

|  |  |
| --- | --- |
 Reference:3 || Sentence | "Furthermore, transfected c-jun, jun-B, jun-D, c-fos, or Fos-B expression vectors partially substitute for TPA and Ca2+ and cooperate with Oct-2 for the transactivation of the combined OAP/octamer cis-element." |
| PMID | 7931079 |
| Year | 1994 |
| Species | Human |
| Journal | J Exp Med |
| RefScore | 0 |
| Source | PArchNLP |
  ||  |  |


---

|  |  |
| --- | --- |
 Reference:4 || Sentence | "Here, we demonstrate that carbonic anhydrase II (CA II), an enzyme necessary for osteoclast activity, is transcriptionally upregulated by c-Fos/AP-1." |
| PMID | 11382925 |
| Year | 2001 |
| Species | Human |
|  | Rat |
| Journal | J Cell Physiol |
| RefScore | 1 |
| Source | PArchNLP |
  |


---

|  |  |
| --- | --- |
